# Advanced glycation end products modulate electrophysiological remodeling of right ventricular outflow tract cardiomyocytes: A novel target for diabetes‐related ventricular arrhythmogenesis

**DOI:** 10.14814/phy2.15499

**Published:** 2022-11-02

**Authors:** Yao‐Chang Chen, Yen‐Yu Lu, Wen‐Shiann Wu, Yung‐Kuo Lin, Yi‐Ann Chen, Shih‐Ann Chen, Yi‐Jen Chen

**Affiliations:** ^1^ Department of Biomedical Engineering National Defense Medical Center Taipei Taiwan; ^2^ Division of Cardiology Sijhih Cathay General Hospital New Taipei City Taiwan; ^3^ School of Medicine, College of Medicine Fu Jen Catholic University New Taipei City Taiwan; ^4^ Department of Cardiology Chi‐Mei Medical Center Tainan Taiwan; ^5^ Taipei Heart Institute, Taipei Medical University Taipei Taiwan; ^6^ Division of Cardiovascular Medicine, Department of Internal Medicine Wan Fang Hospital, Taipei Medical University Taipei Taiwan; ^7^ Division of Cardiology, Department of Internal Medicine, School of Medicine, College of Medicine Taipei Medical University Taipei Taiwan; ^8^ Division of Nephrology Sijhih Cathay General Hospital New Taipei City Taiwan; ^9^ Heart Rhythm Center, Division of Cardiology, Department of Medicine Taipei Veterans General Hospital Taipei Taiwan; ^10^ Cardiovascular Center, Taichung Veterans General Hospital Taichung Taiwan; ^11^ Department of Post‐Baccalaureate Medicine, College of Medicine National Chung Hsing University Taichung Taiwan; ^12^ Graduate Institute of Clinical Medicine, College of Medicine Taipei Medical University Taipei Taiwan; ^13^ Cardiovascular Research Center Wan Fang Hospital, Taipei Medical University Taipei Taiwan

**Keywords:** advanced glycation end products, electrophysiology, right ventricular outflow tract, ventricular arrhythmia

## Abstract

Diabetes mellitus is associated with cardiovascular disease and cardiac arrhythmia. Accumulation of advanced glycation end products closely correlates with cardiovascular complications through mitochondrial dysfunction or oxidative stress and evoke proliferative, inflammatory, and fibrotic reactions, which might impair cardiac electrophysiological characteristics and increase the incidence of cardiac arrhythmia. This study examined the mechanisms how advanced glycation end products may contribute to arrhythmogenesis of right ventricular outflow tract—a unique arrhythmogenic substrate. A whole‐cell patch clamp, conventional electrophysiological study, fluorescence imaging, Western blot, and confocal microscope were used to study the electrical activity, and Ca^2+^ homeostasis or signaling in isolated right ventricular outflow tract myocytes with and without advanced glycation end products (100 μg/ml). The advanced glycation end products treated right ventricular outflow tract myocytes had a similar action potential duration as the controls, but exhibited a lower L‐type Ca^2+^ current, higher late sodium current and transient outward current. Moreover, the advanced glycation end products treated right ventricular outflow tract myocytes had more intracellular Na^+^, reverse mode Na^+^–Ca^2+^ exchanger currents, intracellular and mitochondrial reactive oxygen species, and less intracellular Ca^2+^ transient and sarcoplasmic reticulum Ca^2+^ content with upregulated calcium homeostasis proteins and advanced glycation end products related signaling pathway proteins. In conclusions, advanced glycation end products modulate right ventricular outflow tract electrophysiological characteristics with larger late sodium current, intracellular Na^+^, reverse mode Na^+^–Ca^2+^ exchanger currents, and disturbed Ca^2+^ homeostasis through increased oxidative stress mediated by the activation of the advanced glycation end products signaling pathway.

## Graphical abstract

Diabetes mellitus is associated with cardiac arrhythmia. Advanced glycation end products (AGE) accumulation in diabetes mellitus may elicit mitochondrial dysfunction/oxidative stress generation and evoke proliferative, inflammatory and fibrotic reactions through AGE‐receptor for AGE (RAGE) signaling pathway, which might impair cardiac electrophysiological characteristics and increases the incidence of cardiac arrhythmias. Our result support that AGEs modulate RVOT electrophysiological characteristics through the activation of AGE‐RAGE signaling pathway with ionic currents modulation, increased intracellular and mitochondrial ROS, and disturbed Ca^2+^ homeostasis, which might play a role in diabetes related ventricular arrhythmogenesis and electrical remodeling.

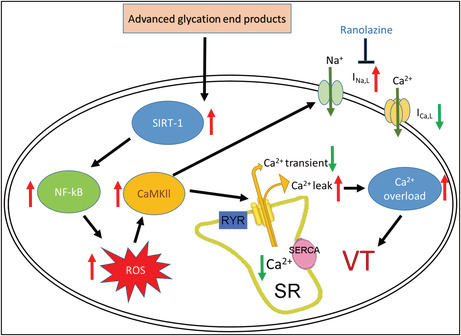

## 1 INTRODUCTION

Diabetes mellitus (DM) is a group of metabolic disorders characterized by high blood sugar levels, which may lead to numerous health complications, such as cardiovascular disease (DeFronzo et al., 2015; Forbes & Cooper, 2013). DM affects the electrical conduction system of the heart and causes atrial and ventricular remodeling, which may result in atrial fibrillation (AF) and ventricular arrhythmia (Agarwal & Singh, 2017; Benjamin et al., 1994; Dahlqvist et al., 2017; Huxley et al., 2011, 2012; Mellbin et al., 2013; Stahn et al., 2014). Patients with DM have a higher risk of AF (Benjamin et al., 1994; Dahlqvist et al., 2017; Huxley et al., 2011, 2012), higher incidence of sudden death, and according to electrocardiographic evidence, a higher propensity of ventricular arrhythmia occurrence than patients without DM (Agarwal & Singh, 2017; Jermendy et al., 1990; Mellbin et al., 2013; Ramirez et al., 2011; Stahn et al., 2014; Veglio et al., 2002). DM may contribute to ventricular arrhythmia because of the shared mechanisms linking DM with AF.

Hyperglycemia results in an excessive formation of advanced glycation end products (AGEs), which can further deteriorate the pathology of diabetes. AGEs are a heterogeneous, complex group of compounds that are formed mainly through glycation (Luevano‐Contreras & Chapman‐Novakofski, 2010), which occurs when reducing sugar reacts in a nonenzymatic manner (Maillard reaction) with amino acids in proteins, lipids, or DNA as a result of chronic hyperglycemia. The accumulation of AGEs in patients with DM is associated with asymptomatic diastolic and systolic cardiac dysfunction, and the development and progression of heart failure (Berg et al., 1999; Hartog et al., 2007). The AGE receptor (RAGE) for the AGE signaling pathway is involved in the pathogenesis of diabetes and diabetic complications. The level of circulating soluble RAGEs is correlated with the severity of cardiac dysfunction (Raposeiras‐Roubín et al., 2010; Wang et al., 2011). The AGEs/RAGE axis can elicit mitochondrial dysfunction and generate reactive oxygen species (ROS), which in turn activate a nuclear factor kappa B (NFκB) cascade (Gloire et al., 2006; Wang et al., 2015; Wautier et al., 2001; Yamagishi et al., 2015). Augmented systemic oxidative stress is associated with cardiac electrical and structural remodeling, which may impair Na^+^, potassium (K^+^), and calcium (Ca^2+^) channels and Na–Ca exchanger (NCX) activity, thus leading to gap junction remodeling, decreased action potential (AP) amplitude (APA) and AP duration (APD), and increased incidence of cardiac arrhythmia in animal models (Dong & Ren, 2014; Drolet et al., 2007; Jeong et al., 2012).

The right ventricular outflow tract (RVOT) is an arrhythmogenic substrate with a unique electropharmacologic profile (Maury, 2011). Premature ventricular complexes, and ventricular arrhythmia, such as idiopathic ventricular tachycardia (VT), Brugada syndrome, arrhythmogenic right ventricular dysplasia, and torsade de pointes, frequently originate from the RVOT (Kim et al., 2007; Morita et al., 2003; Tsai et al., 1997). RVOT cardiomyocytes have a longer APD and greater Ca^2+^ content than RV apex cardiomyocytes, which might contribute to RVOT arrhythmogenesis (Lu et al., 2014). Additionally, the larger *I*_Na‐late_ in RVOT cardiomyocytes may alter the rate of Na^+^ extrusion/Ca^2+^ entry and lead to intracellular Ca^2+^ overload, resulting in the genesis of early afterdepolarization (EAD) (Lu et al., 2014).

However, knowledge of the role of AGEs in RVOT arrhythmogenesis remains limited. The cardiac effects of AGEs may contribute to the pathogenesis of RVOT arrhythmogenesis. Therefore, this study examined the role of AGEs in RVOT arrhythmogenesis, which may underlie DM‐related ventricular arrhythmogenesis.

## 2 MATERIALS AND METHODS

### 2.1 Rabbit RVOT tissue preparation

This investigation was approved by a local ethics review board (No. IACUC‐20‐025) and conformed to the institutional *Guide for the Care and Use of Laboratory Animals* and the *Guide for the Care and Use of Laboratory Animals, 8th Edition*, published by the United States National Institutes of Health (Washington DC, 2011). Using a precision vaporizer, we anesthetized male rabbits (weighing approximately 1.5–2 kg) with an overdose of isoflurane (5% O_2_) while restraining them in a dorsal recumbent position. The lack of a corneal reflex and motor response to pain stimuli with a scalpel tip confirmed the adequacy of the anesthesia. The RVOT (surrounded superiorly and inferiorly by the supraventricular crest and pulmonary valve, and was excised within 5 mm below the pulmonary valve) and RVA (area within 5 mm of the RVA) were excised from all rabbits after euthanasia and prepared for analysis (Lu et al., 2014). RVOT and RVA tissue were treated in the 100 μg/ml BSA (BioVision) or 100 μg/ml AGE (BioVision) +100 μg/ml BSA for 4–6 h prior to subsequent electrophysiological and pharmacological studies.

### 2.2 Electrophysiological and pharmacological studies of RVOT and RVA

The tissue preparations were bathed in Tyrode's solution (B5), which comprised 137 mM NaCl, 4 mM KCl, 15 mM NaHCO_3_, 0.5 mM NaH_2_PO_4_, 0.5 mM MgCl_2_, 2.7 mM CaCl_2_, and 11 mM dextrose, at 37°C. The tissues were superfused at a constant rate (3 ml/min) with Tyrode's solution, which was saturated with a gas mixture of 97% O_2_ and 3% CO_2_. The transmembrane APs of the RVOT tissues were recorded using machine‐pulled glass capillary microelectrodes filled with 3 M KCl, and the tissue preparations were connected to a World Precision Instrument model WPI Duo 773 electrometer. The electrical and mechanical events were simultaneously displayed on a Gould 4072 oscilloscope and Gould TA11 recorder. Electrical stimuli were applied using a Grass S48 stimulator through a Grass SIU5B stimulus isolation unit. Burst firing was defined as the occurrence of an accelerated spontaneous AP with sudden onset and termination. After the control and AGE (100 μg/ml)‐treated RVOT or RVA tissues were analyzed and the effects of treatment during high‐frequency burst pacing (20 Hz) for 1 s. An inhibitor of late sodium current—ranolazine (10 μM) was added to observe burst firing in RVOT. Sustained VT and nonsustained VT were defined as ventricular bursting beating of more and less than 30 s, respectively. Spontaneous activity was defined as the constant occurrence of spontaneous APs in the absence of electrical stimuli. Burst firing was defined as accelerated spontaneous activity with a beat faster than the basal beating activity and sudden onset and termination. Triggered activity was defined as premature beats or delayed afterdepolarizations (DADs). EAD and DAD were defined as the presence of a spontaneous depolarization of the impulse after complete repolarization.

### 2.3 Isolation of RVOT and RVA cardiomyocytes and a whole‐cell patch clamp

Single cardiomyocytes from rabbit RVOTs and RVAs were enzymatically dissociated through a procedure described by Lu et al. (2014) and were used for this study. After euthanasia, the rabbits' hearts were excised and mounted on a Langendorff apparatus to be superfused in an antegrade manner at 37°C with oxygenated normal Tyrode's solution (B1). After the blood was removed from the hearts, the perfusate was replaced with oxygenated Ca^2+^‐free Tyrode's solution containing 300 units/ml of collagenase type I (Sigma‐Aldrich) and 0.25 units/ml protease type XIV (Sigma‐Aldrich) for 8–12 min. The RVOT was excised and gently shaken in 50‐ml of Ca^2+^‐free oxygenated Tyrode's solution until single cardiomyocytes were obtained. The solution was then gradually replaced by normal oxygenated Tyrode's solution. The cardiomyocytes stabilized in a bath for at least 30 min before the experiments commenced. Isolated RVOT cardiomyocytes were treated in the BSA (100 μg/ml) or AGE (100 μg/ml) + BSA (100 μg/ml) for 4–6 h prior to ionic currents, Ca^2+^ transients, intracellular ROS, Na^+^, and Western blot analysis. The detailed contents of external (Tyrode's) and intracellular (pipette) solutions used in this study were summarized in Table 1.

**TABLE 1:** External (Tyrode's) and intracellular (pipette) solutions used in this study.

	Pipette solutions, mmol/L						Bath solutions, mmol/L				
	P_1_	P_2_	P_3_	P_4_	P_5_	P_6_	B_1_	B_2_	B_3_	B_4_	B_5_
NaCl		20	5	10			137	5	130	140	137
CaCl_2_		1.75				0.36	1.8	1.8	1	2	2.7
MgCl_2_	1	0.4			1	5	0.5	2	1	1	0.5
KCl					20	120	5.4				4
CsCl	130	110	133	130				133	5		
TEACl		20	20								
MgATP	5	5	5	5	5						
LiGTP					0.1						
NaGTP	0.1					0.25					
K aspartate					110	5					
Na_2_ phosphocreatine	5				5	5					
EGTA	10		10	5	0.5	5					
BAPTA		5									
HEPES	10	10	5	5	10	5	10	5	10	5	
glucose		5		5		5	11	5	10	10	
Nifedipine								0.002			
Niflumic acid										0.1	
Nitrendipine										0.01	
Strophanthidin										0.01	
NaH_2_PO_4_											0.5
NaHCO_3_											15
dextrose											11
pH	7.2	7.25	7.3	7.3	7.2	7.2	7.4	7.3	7.4	7.4	7.4
	(CsOH)	(CsOH)	(CsOH)	(NaOH)	(KOH)	(KOH)	(NaOH)	(NaOH)	(NaOH)	(NaOH)	(NaOH)

Abbreviation: TEACl, tetraethylammonium chloride.

Consistent with the procedure described by Lu et al. (2014), a whole‐cell patch clamp was used to record ionic currents and APs on isolated RVOTs with an Axopatch 1D amplifier (Axon Instruments) at 35°C ± 1°C. Borosilicate glass electrodes (o.d., 1.8 mm) were used, with tip resistances of 3–5 MΩ. Before the formation of the membrane‐pipette seal, the tip potentials were zeroed in the solution B1. The junction potentials (9 mV) measured from the differences between the bath (B1) and pipette (P1) solutions were corrected for the action potential (AP) recordings. The ionic currents were recorded approximately 3–5 min after rupture or perforation to avoid the decay of ion channel activity over time. At the beginning of each experiment, a small hyperpolarizing step was delivered from a holding potential of −50 mV to a test potential of −55 mV for 80 ms. The area under the capacitive current curve was divided by the applied voltage step to obtain the total cell capacitance. Typically, 60%–80% of the series resistance (Rs) was electronically compensated for. AP parameters were resting membrane potential (RMP), APA, and APD at 20%, 50%, and 90% repolarization of the amplitude (APD_20_, APD_50_, and APD_90_).

The APs was recorded in the pipette solution P5. *I*_Ca‐L_, NCX currents, *I*_Na_, *I*_Na‐late_, *I*_to_ and *I*_K1_ were recorded in the pipette solutions P1, P2, P3, P4 and P5, respectively.

The *I*_Na_ was recorded during depolarization from a holding potential of −120 mV to the test potentials, which ranged from −90 to 0 mV in 5‐mV steps for 40 ms at a frequency of 3 Hz, at room temperature (25°C ± 1°C) in the bath solution B2.

The *I*_Na‐late_ was recorded at room temperature in the bath solution B3 using a step‐ramp protocol (−100 mV stepped to +20 mV for 100 ms, then ramped back to −100 mV over 100 ms). An equilibration period for dialysis was allowed for the adequate clamping of the cell currents. The *I*_Na‐late_ was measured as the tetrodotoxin (30 μM)‐sensitive portions of the current traces obtained when the voltage was ramped back to −100 mV, a process described by Lin et al. (2012).

The *I*_Ca‐L_ was measured as an inward current during depolarization from a holding potential of −50 mV to test potentials ranging from −40 to 60 mV in 10‐mV steps for 300 ms at a frequency of 0.1 Hz with constant voltage using a perforated patch clamp with amphotericin B. NaCl and KCl in the bath solution B1 were replaced with tetraethylammonium chloride and CsCl, respectively. The current recorded at the peak was selected to represent the *I*_Ca‐L_, which was measured as amplitudes of the *I*_Ca‐L._

The NCX current was elicited using test pulses between −100 and 100 mV from a holding potential of −40 mV for 300 ms at a frequency of 0.1 Hz. The NCX current was measured as 10‐mM nickel‐sensitive currents recorded under control conditions; following the addition of Ni^2+^ through digital subtraction at the end portion, the NCX current was selected to represent the Na–Ca exchange, which was measured as amplitudes of the NCX current in the bath solution B4.

The transient outward K^+^ current (*I*_to_) was examined through a double‐pulse protocol. A 30‐ms prepulse from −80 to −40 mV was used to inactivate the Na^+^ channels; this was followed by a 300‐ms test pulse to 60 mV in 10‐mV steps at a frequency of 0.1 Hz. CdCl_2_ (200 μM) was added to the bath solution B1 to inhibit the *I*_Ca‐L_. The *I*_to_ was measured as the difference between the peak outward current and the steady‐state current. The inward rectifier K^+^ current (*I*_K1_) was activated from −40 mV to the test potentials, ranging from −20 to 120 mV, in 10‐mV steps for 1 s at a frequency of 0.1 Hz under an infusion of 200 μM CdCl_2_ and 2 mM 4‐aminopyridine in the bath solution B1. The amplitudes of the *I*_K1_ were measured as 1 mM barium‐sensitive currents.

The rapid delayed rectifier K^+^ current (*I*_Kr‐tail_) was measured as the outward peak tail current density following a 3‐s prepulse from a holding potential of −40 mV to a voltage between −40 and 60 mV in 10‐mV steps at a frequency of 0.1 Hz in the presence of 30 μM chromanol 293B and 200 μM CdCl_2_ in the Ca^2+^‐free normal Tyrode's solution (the bath solution B1). Micropipettes were filled with the pipette solution P6.

### 2.4 Measurement of Ca^2+^ transients and intracellular Ca^2+^

With reference to the experimental design developed by Suenari et al. (2011), fluorescent Ca^2+^ (10 μmol/L) fluo‐3/AM was loaded onto the control and AGE‐treated RVOT cardiomyocytes for 30 min at room temperature. After intracellular hydrolysis of fluo‐3/AM was conducted for 30 min, excess extracellular dye was removed by changing the bath solution B1. Fluo‐3 fluorescence (F) was excited with the 488‐nm line of an argon ion laser and the emission was recorded at >515 nm. We repeatedly scanned cells at 2‐ms intervals for line scan imaging (8‐bit). F imaging was performed with a laser scanning confocal microscope (Zeiss LSM 510; Carl Zeiss) and an inverted microscope (Axiovert 100; Carl Zeiss). To exclude variations in the F intensity caused by various volumes of injected dye and to correct for variations in dye concentrations, the fluorescent signals were calculated through the normalization of the F against the baseline F (F_0_), and reliable information about transient intracellular Ca^2+^ (Ca^2+^_i_) changes from baseline values [(F − F_0_)/F_0_] were thus obtained. The intracellular Ca^2+^ changes, including transient Ca^2+^_i_, peak systolic Ca^2+^_i_, and diastolic Ca^2+^_i_, were obtained during a 1‐Hz field stimulation with 10‐ms twice‐threshold‐strength square‐wave pulses. After the addition of 20 mM caffeine after electric stimulation at 1 Hz for at least 30 s, the estimated sarcoplasmic reticulum (SR) Ca^2+^ content, used as the total SR Ca^2+^ content, was measured from the peak amplitude of the caffeine‐induced Ca^2+^_i_ transients (Suenari et al., 2011). To minimize cell motion during contraction, the scan line was positioned along the short axis (transversal scan) in the central region of the cell avoiding the nucleus. After steady‐state Ca^2+^ transients were achieved with repeated pulses (1 Hz for 5 s).

### 2.5 Measurement of intracellular ROS and Na^+^

We used CellROX Green (Life Technologies) to assess cytosolic ROS production and Asante NaTRIUM Green‐2 AM (Teflabs) to evaluate cytosolic Na^+^ (Na^+^_i_) concentrations of control and AGE‐treated RVOT cardiomyocytes. Experiments were performed using a laser scanning confocal microscope (Zeiss LSM 510) and inverted microscope (Axiovert 100) with a 63 × 1.25 numerical aperture oil immersion objective, as described by Viatchenko‐Karpinski et al. (2014). Cardiomyocytes were maintained in normal Tyrode's solution of 137 mM NaCl, 5.4 mM KCl, 1.8 mM CaCl_2_, 0.5 mM MgCl_2_, and 10 mM HEPES with an appropriate fluorescent dye of 10 μmol/L CellROX Green and 5 μmol/L Asante NaTRIUM Green‐2 am. The CellROX Green or Asante NaTRIUM Green‐2 AM were excited at 488 nm, and F signals were acquired at wavelengths of >505 nm in the XY mode of the confocal system. Fluorescent images were analyzed using Image‐Pro Plus 6.0 and Sigma Plot 12, as described by Suenari et al. (2011). Fluorescent signals were calculated by normalizing F against F_0_, thus obtaining reliable information on transient intracellular ROS changes or intracellular Na^+^ (Na^+^_i_) changes from baseline values [(F − F_0_)/F_0_].

### 2.6 Western blot analysis

Control and AGE (100 μg/ml)‐treated RVOT cardiomyocytes were centrifuged and washed with cold phosphate‐buffered saline and lysed on ice for 30 min in a RIPA buffer comprising 50 mM Tris (pH 7.4), 150 mM NaCl, 1% NP40, 0.5% sodium deoxycholate, 0.1% sodium dodecylsulfate (SDS), and protease inhibitor cocktails (Sigma‐Aldrich). The protein concentration was determined with a Bio‐Rad protein assay reagent (Bio‐Rad). Proteins were separated in 4%–12% SDS‐polyacrylamide gel electrophoresis (PAGE) under reducing conditions and electrophoretically transferred into an equilibrated polyvinylidene difluoride membrane (Amersham Biosciences). All blots were probed with primary antibodies against RAGE, Sirtuin1 (SIRT1), pSIRT1, NFκB, inositol 1,4,5‐trisphosphate receptor (IP3R) and pIP3R, P38, protein kinase C (PKC; Swant, Switzerland), SR/endoplasmic reticulum (SR/ER) (Ca^2+^ATPase 2a [SERCA2a]), Ca^2+^/calmodulin‐dependent protein kinase II (CaMKII), pCaMKII, ryanodine receptors (RYR), pRYR S2808, pRYR S2814, Cav1.2, NCX, and beta‐actin. All secondary antibodies were conjugated with horseradish peroxidase. Moreover, all bound antibodies were detected using an enhanced chemiluminescence detection system and analyzed using AlphaEaseFC software (Alpha Innotech). All targeted bands were normalized to beta‐actin to confirm equal protein loading.

### 2.7 Statistical analysis

All continuous variables are expressed as the mean ± standard error of the mean. The electrophysiological characteristics of the RVOT without or with AGEs were compared using a one‐way or two‐way analysis of variance (ANOVA) with a post hoc Tukey's analysis by Sigmaplot version 12.0 for multiple comparisons. The ionic currents of RVOT cardiomyocytes without or with AGEs were compared using either the Wilcoxon rank‐sum test or unpaired *t* test, depending on the results of the normality test. Nominal variables were compared using a chi‐square analysis with Fisher's exact test, and a *p* value of <0.05 was considered statistically significant.

## 3 RESULTS

### 3.1 Effects of AGEs on electrical activity and ionic currents

**FIGURE 1:**
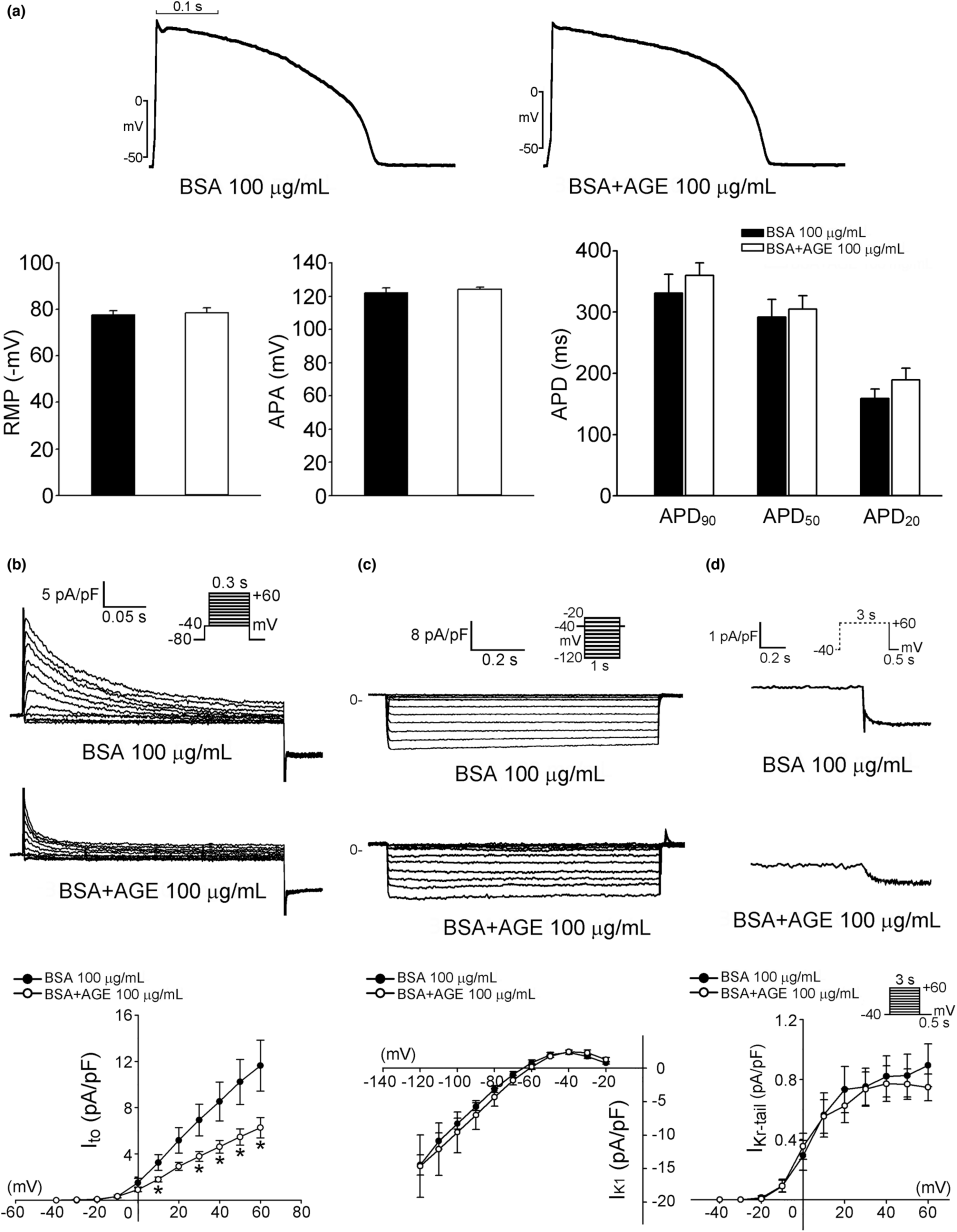
Effects of advanced glycation end products (AGEs) on electrical activity in the right ventricular outflow tract (RVOT) and effects of AGEs on potassium currents of RVOT cardiomyocytes. (a) Examples and average data of RMP, APA, and APD_90_, APD_50_, and APD_20_ in AGE 100 μg/ml superfused (*n* = 11, *N* = 6) and control (*n* = 14, *N* = 8) RVOT myocytes. (b) Example traces of current tracings and the I–V relationship of *I*_to_ in RVOT myocytes without (control, *n* = 9, *N* = 3) or with (*n* = 10, *N* = 6) AGE 100 μg/ml. (c) Example traces of current tracings and the I–V relationship of *I*_K1_ in RVOT myocytes without (control, *n* = 10, *N* = 4) or with (*n* = 8, *N* = 4) AGE 100 μg/ml. (d) Example traces of current tracings and the I–V relationship of delayed rectifier current (*I*_Kr‐tail_) in RVOT myocytes without (control, *n* = 10, *N* = 4) or with (*n* = 11, *N* = 5) AGE 100 μg/ml. *p < 0.05. Inset depicts the various clamp protocols. *n*, cell number; *N*, animal number

**FIGURE 2:**
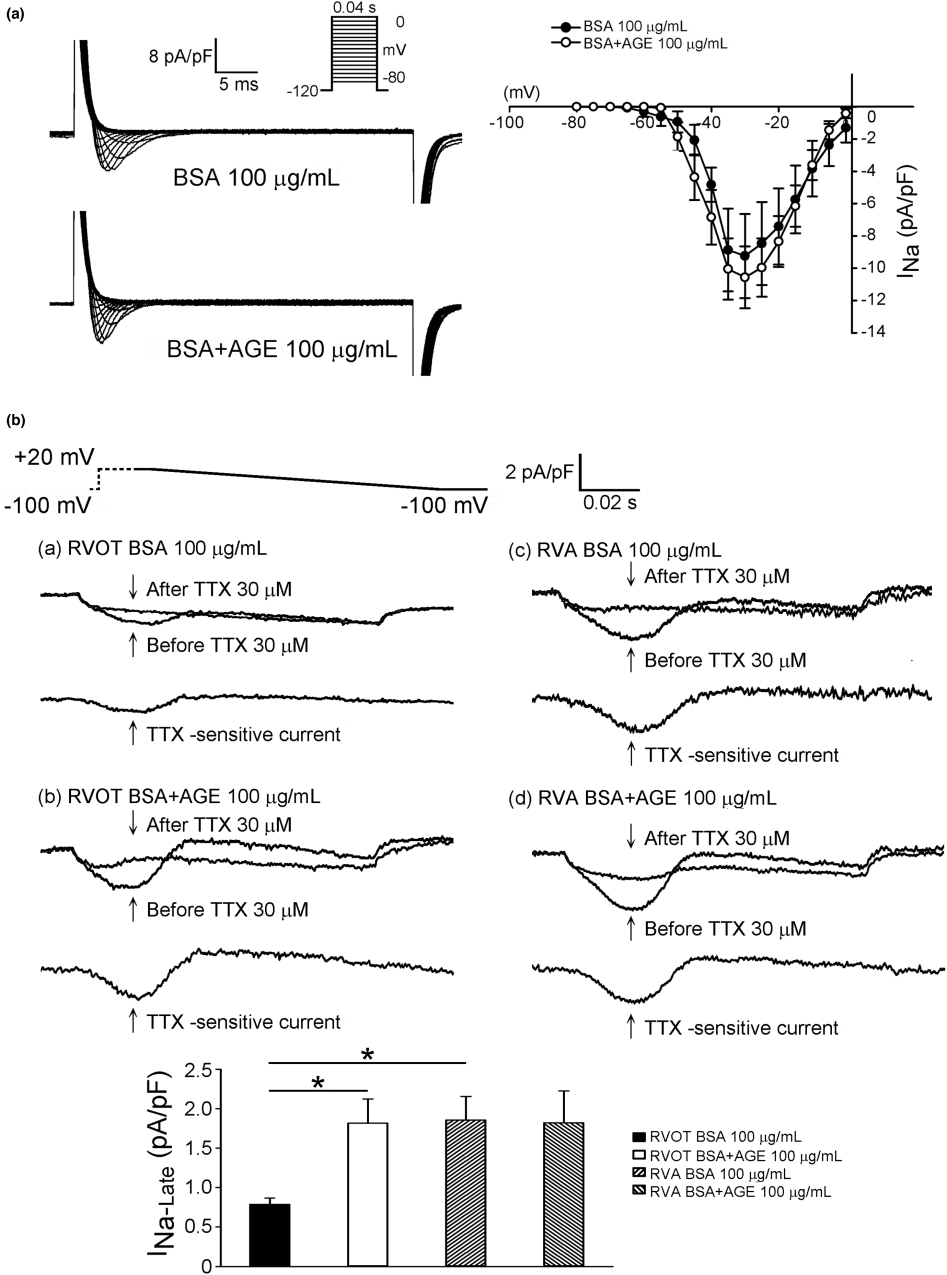
Effects of AGEs on *I*_Na_ and *I*_Na‐late_ in RVOT and RVA cardiomyocytes. (a) Current traces and I–V relationship of the *I*_Na_ in RVOT myocytes without (control, *n* = 11, *N* = 6) or with (*n* = 13, *N* = 6) AGE 100 μg/ml. (b) Current traces and the average data of *I*_Na‐late_ in RVOT and RVA myocytes without (control, *n* = 10, *N* = 7) or with (*n* = 13, *N* = 6) AGE 100 μg/ml. *p < 0.01. Inset depicts the various clamp protocols. *n*, cell number; *N*, animal number

**FIGURE 3:**
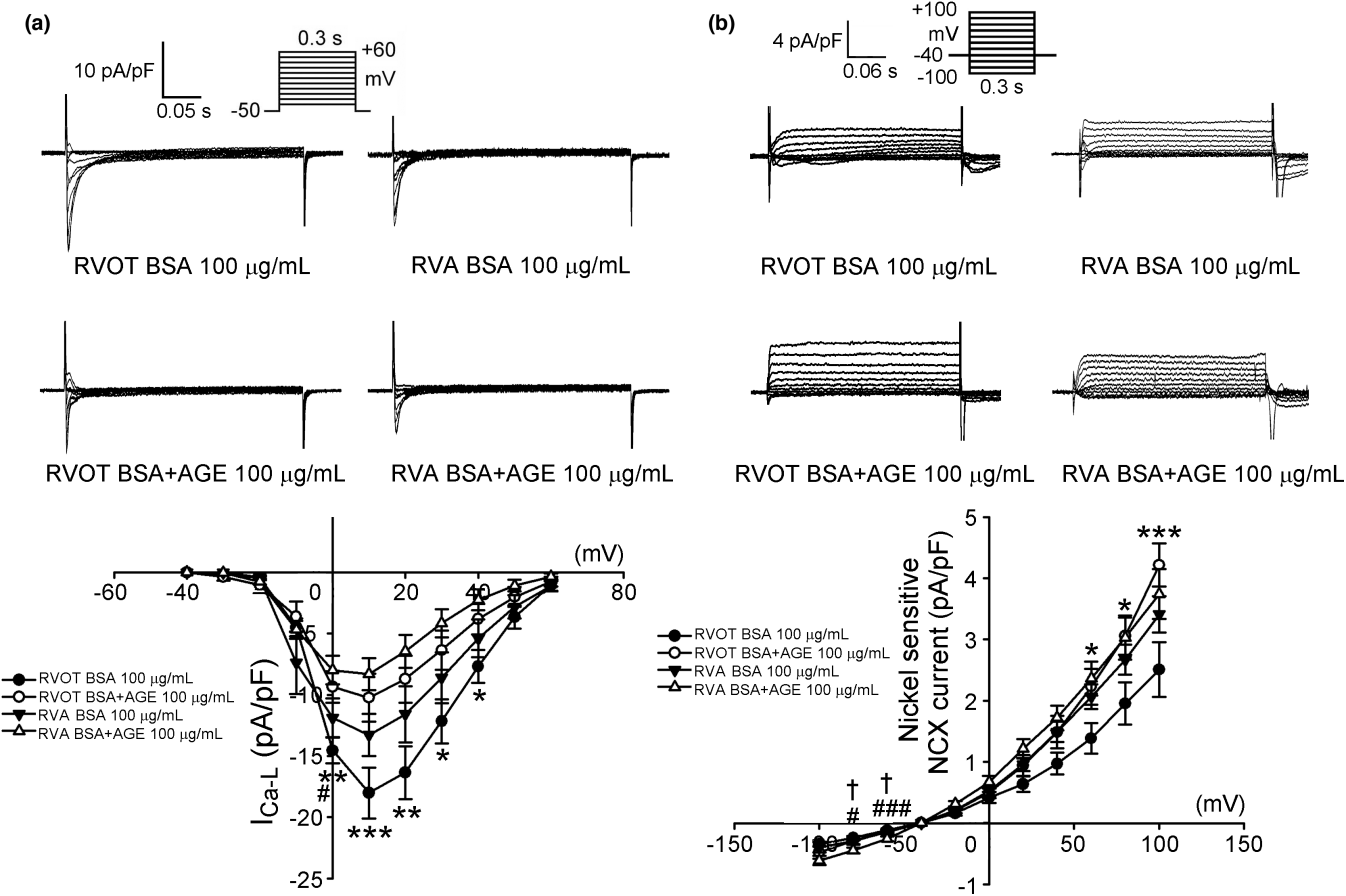
Effects of AGEs on *I*_Ca‐L_ and the nickel‐sensitive Na^+^–Ca^2+^ exchanger (NCX) current in RVOT and RVA cardiomyocytes. (a) Current traces and I–V relationship of the *I*_Ca‐L_ in RVOT and RVA myocytes without (control, *n* = 9, *N* = 4; *n* = 6, *N* = 3) or with (*n* = 7, *N* = 5; *n* = 9, *N* = 3) AGE 100 μg/ml. (b) Example traces of current tracings and the I–V relationship of the NCX current in RVOT and RVA myocytes without (control, *n* = 9, *N* = 6; *n* = 11, *N* = 4) or with (*n* = 9, *N* = 6; *n* = 10, *N* = 4) AGE 100 μg/ml. *p < 0.05, **p < 0.01, RVOT with and without AGE. #*p* < 0.05, ##*p* < 0.01, RVA with and without AGE. ^†^ *p* < 0.05, RVOT vs. RVA with AGE. Inset depicts the various clamp protocols. *n*, cell number; *N*, animal number

Representative tracings of RVOT cardiomyocytes without or with AGEs (100 μg/ml) are presented in Figure 1a. Treatment with AGEs did not alter APD_90_, APD_50_, APD_20_, RMP, or APA, which was inconsistent with clinically observed QT interval prolongation in patients with DM. The reduction of the *I*_to_ following treatment with AGEs is depicted in Figure 1b. Moreover, as illustrated in Figure 1c,d, the *I*_K1_ and *I*_Kr‐tail_ were not influenced by AGE treatment. The effect of AGEs on the sodium current is depicted in Figure 2, and the *I*_Na_ remained unchanged regardless of the presence of AGEs (Figure 2a). However, *I*_Na‐late_ was significantly increased in RVOT cardiomyocytes treated with AGEs, but was not changed in RVA cardiomyocytes treated with AGEs (Figure 2b). The change in calcium currents following AGE treatment is depicted in Figure 3; the AGEs significantly reduced *I*_Ca‐L_ in RVOT, but only partially reduced *I*_Ca‐L_ in RVA (Figure 3a). The AGE treatment primarily segmented the NCX current in reverse mode, and on the contrary, in the forward mode in RVA. (Figure 3b).

### 3.2 Effects of AGEs on intracellular ROS, sodium homeostasis, calcium homeostasis, and AGE–RAGE signaling pathway

**FIGURE 4:**
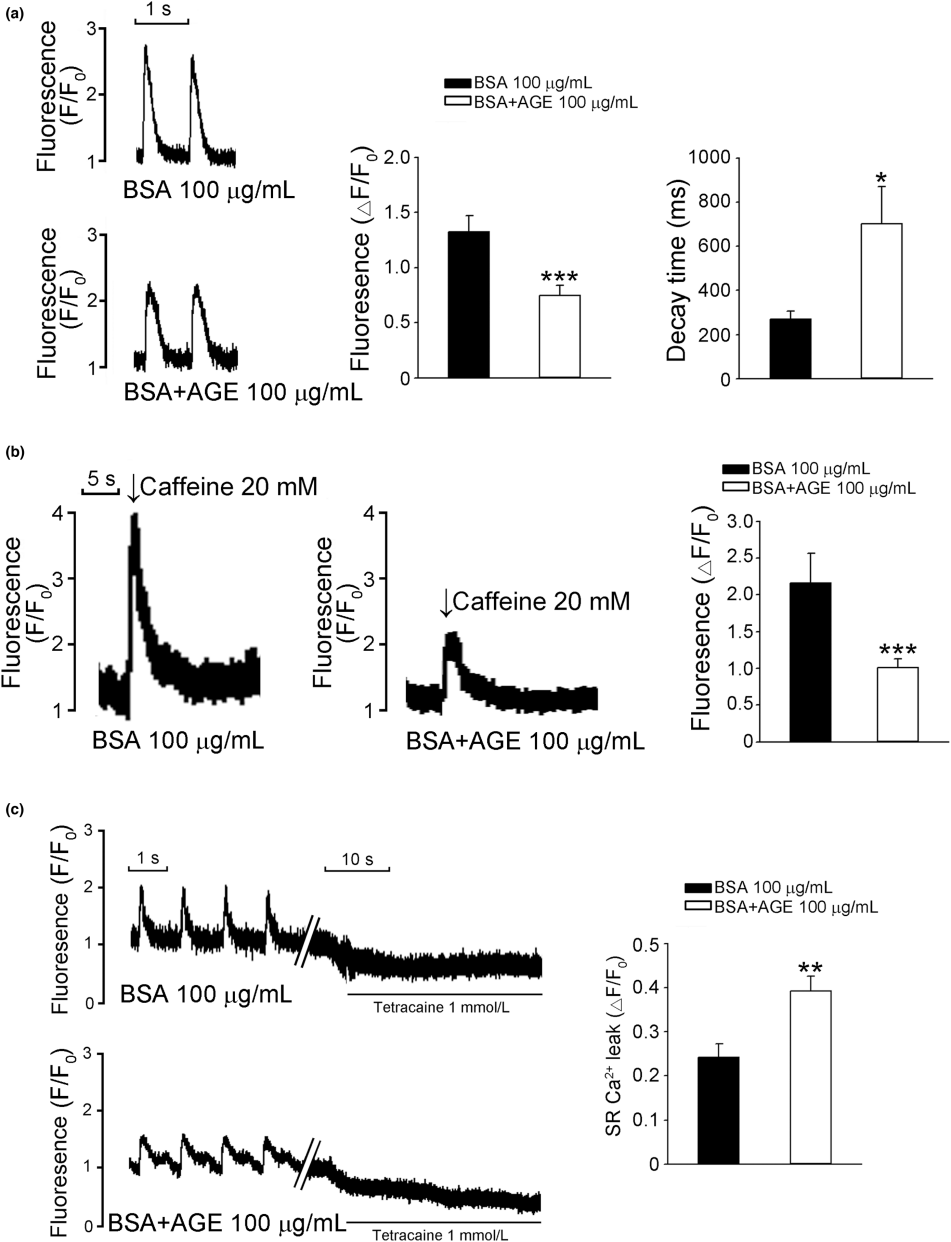
Effects of AGEs on calcium transients, calcium induced calcium transient (sarcoplasmic reticulum [SR] Ca^2+^ contents), and calcium leak in RVOT cardiomyocytes. (a) Example traces and average data of calcium transients in RVOT myocytes without (control, *n* = 13, *N* = 4) or with (*n* = 15, *N* = 5) AGE 100 μg/ml. (b) Example traces and average data of SR Ca^2+^ contents in RVOT myocytes without (control, *n* = 12, *N* = 4) or with (*n* = 16, *N* = 5) AGE 100 μg/ml. (c) Example traces and average data of Ca^2+^ leak in RVOT myocytes without (control, *n* = 8, *N* = 3) or with (*n* = 13, *N* = 5) AGE 100 μg/ml. *p < 0.05, **p < 0.01, ***p < 0.005. These results are representative of data obtained from myocytes in separate control and AGE‐treated populations. *n*, cell number; *N*, animal number

**FIGURE 5:**
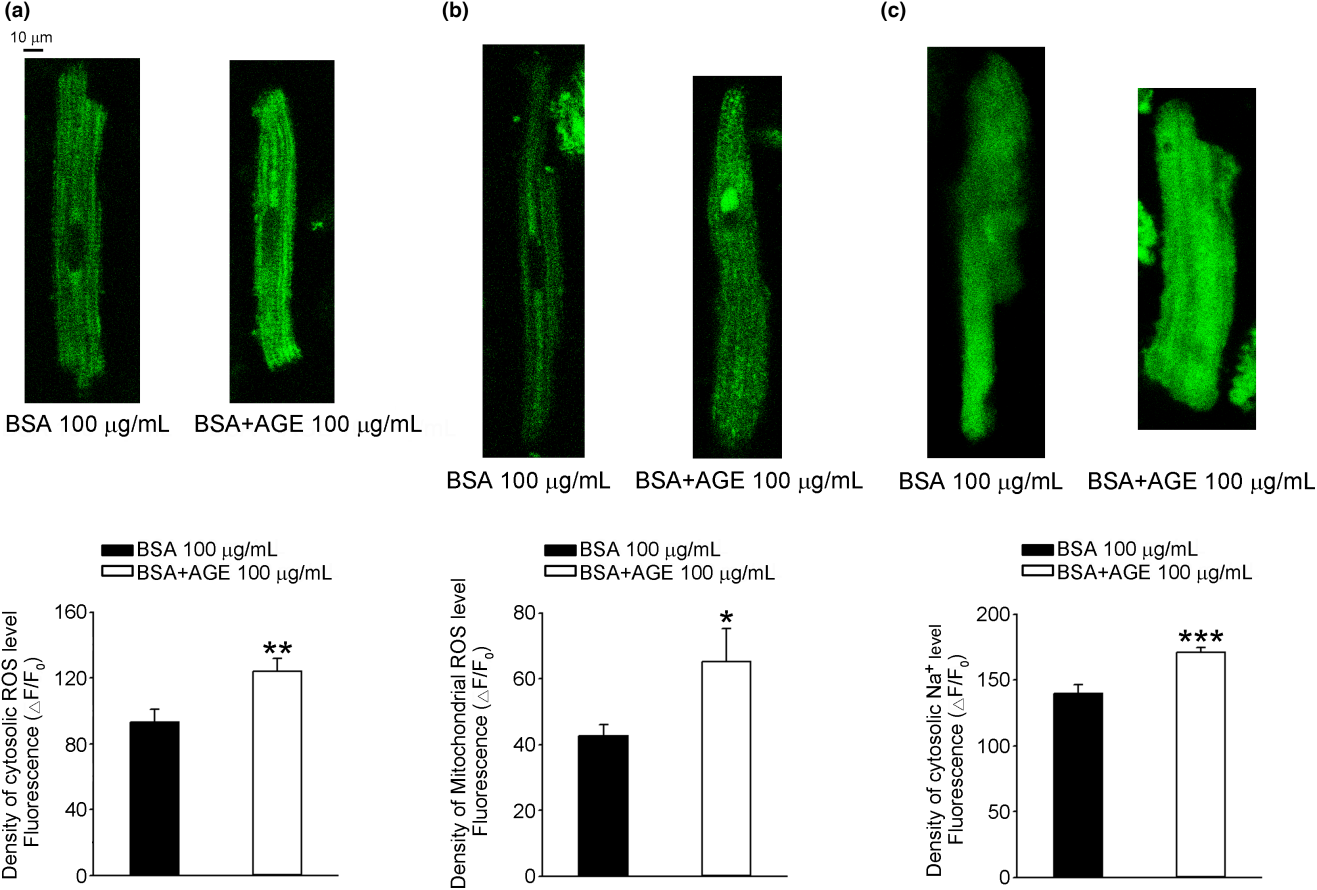
Effects of AGEs on cytosolic reactive oxygen species (ROS), mitochondrial ROS, and cytosolic sodium in isolated RVOT cardiomyocytes. The F intensity compared RVOT cardiomyocytes with AGEs or without (control). **(**a) Examples and average data of cytosolic ROS in RVOT myocytes without (control, *n* = 43) or with (*n* = 30) AGE 100 μg/ml. (b) Example traces and average data of mitochondrial ROS in RVOT myocytes without (control, *n* = 17) or with (*n* = 21) AGE 100 μg/ml. (c) Examples and average data of cytosolic sodium in RVOT myocytes without (control, *n* = 16) or with (*n* = 38) AGE 100 μg/ml. *p < 0.05, **p < 0.01, ***p < 0.005. *n*, cell number

**FIGURE 6:**
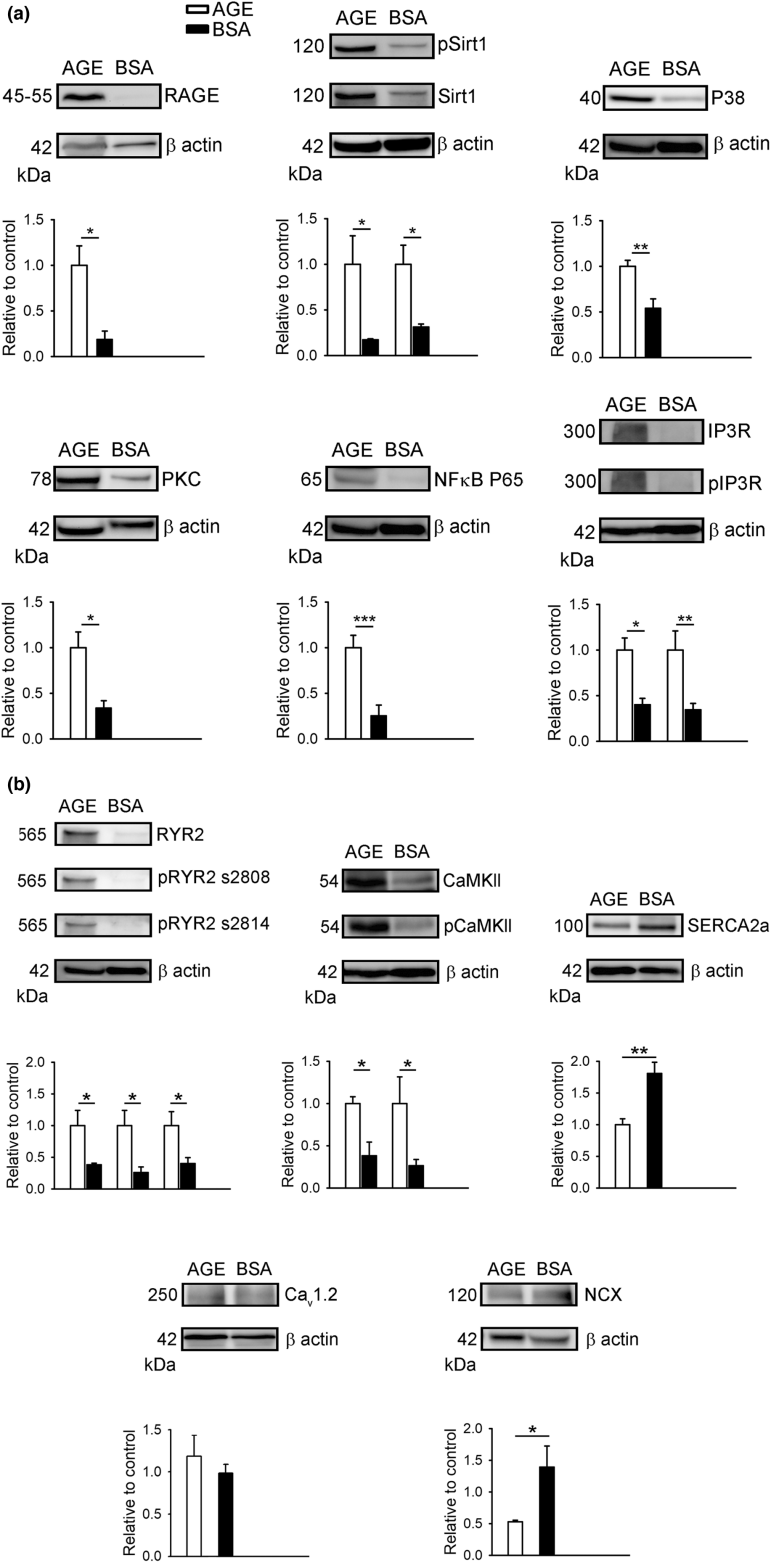
Effects of AGEs on the protein expression of AGE–RAGE and related signaling pathways and the effect of AGE on calcium homeostasis–related proteins of RVOT. (a) Examples and average data (relative to ß Actin) of Western blot of RAGE, NFκB P65, PKC, P38, IP3R, pIP3R, pSirt1, and Sirt1 in RVOT myocytes without (control, *N* = 4) or with (*N* = 4) AGE 100 μg/ml. (b) Examples and average data (relative to ß Actin) of Western blot of RYR2, pRYR2 s2808, pRYR2 s2814, CaMKII, pCaMKII, and SERCA2a in RVOT myocytes without (control, *N* = 4) or with (*N* = 4) AGE 100 μg/ml. *p < 0.05, **p < 0.01, ***p < 0.005. *N*, animal number

We used fluorescent fluo‐3/AM dye to evaluate the effects of AGEs on calcium homeostasis and determined that AGE 100 μg/ml reduced Ca^2+^_i_ transients and SR Ca^2+^ content with a prolongation of decay time in RVOT cardiomyocytes (Figure 4a,b). Despite the reduction in Ca^2+^_i_ transients and SR Ca^2+^ content in RVOT cardiomyocytes, the AGE treatment significantly increased the SR Ca^2+^ leak (Figure 4c). AGE treatment significantly increased the cytosolic ROS (Figure 5a), mitochondrial ROS (Figure 5b), and cytosolic sodium concentration (Na^+^_i_) of RVOT cardiomyocytes (Figure 5c). Compared with the control RVOT cardiomyocytes, the RVOT cardiomyocytes treated with AGE 100 μg/ml exhibited upregulated RAGE, NF_ƙ_B P65, PKC, P38, IP3R, pIP3R, pSirt1, and Sirt1 in the AGE–RAGE–related signaling pathway (Figure 6a). AGEs increased RYR2, pRYR2 s2808, pRYR2 s2814, CaMKII, and pCaMKII but reduced SERCA2a in the calcium homeostasis–related proteins of RVOT cardiomyocytes (Figure 6b).

### 3.3 Effects of AGEs on ability of RVOT to induce ventricular arrhythmia

**FIGURE 7:**
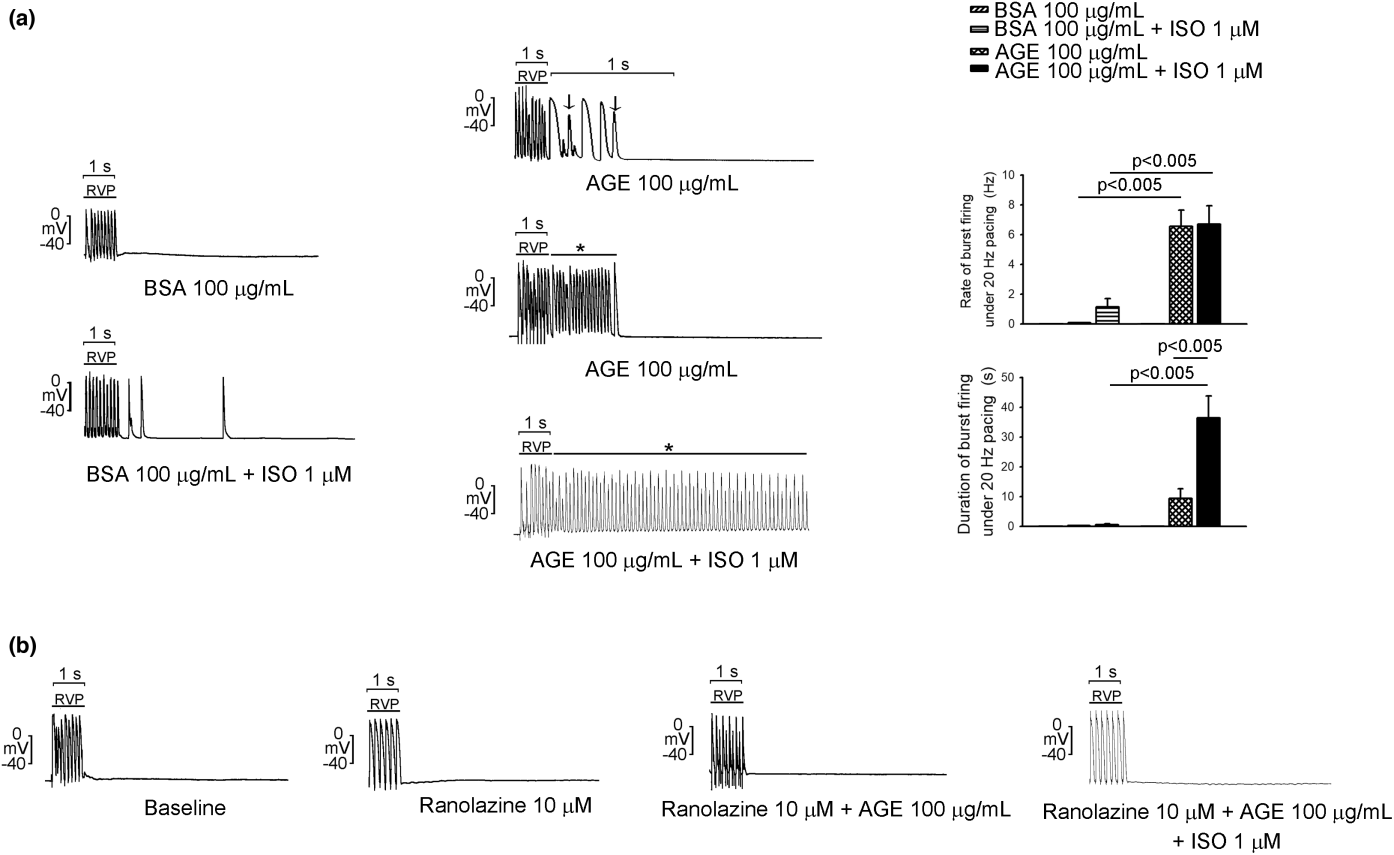
Effects of AGEs on the ability of RVOT to induce ventricular arrhythmia. (a) Examples of burst pacing on RVOT at baseline, after bovine serum albumin, and after isoproterenol (ISO) 1 μM (*N* = 7). Examples of burst pacing on RVOT at baseline, after AGE 100 μg/ml, and after AGE 100 μg/ml + ISO 1 μM (*N* = 7). (b) Examples of burst pacing on RVOT at baseline, after ranolazine 10 μM, after ranolazine 10 μM + AGE 100 mg/ml, and after ranolazine 10 μM + AGE 100 μg/ml + ISO 1 μM (*N* = 5). *N*, animal number

Representative tracings of RVOT with and without AGE 100 μg/ml and the induction of ventricular arrhythmia are displayed in Figure 7. Burst pacing on an RVOT tissue strip could not induce ventricular arrhythmia at baseline or after bovine serum albumin treatment (Figure 7a). After infusion with 1 μM isoproterenol, burst pacing produced sporadic ventricular beats in 4 of 7 RVOT tissue strips (0% vs. 57.1%, *p* < 0.01, Figure 7a) and induced burst firing of ventricular arrhythmia after AGE 100 μg/ml incubation (0% vs. 100%, *p* < 0.005), which was further sustained after the administration of 1 μM isoproterenol (9.4 ± 3.2 s vs. 36.4 ± 7.4 s, *p* < 0.005; Figure 7a). Furthermore, the 10 μM ranolazine infusion stopped the induction of the ventricular arrhythmia burst firing with and without AGE (0% vs. 100%, *p* < 0.005) and/or isoproterenol (0% vs. 57.1%, *p* < 0.05, Figure 7b). There were EADs (4/7, 57.1%) and DADs (1/7, 14%) after AGE treatment compared with EADs (0/7, 0%) and DADs (0/7, 0%) after BSA in intact RVOT strips, however, lack of statistical significance. There was no EAD, DAD, or burst firing induced ventricular arrhythmia in RVA without or with AGE 100 μg/ml incubation (0%, *N* = 5, supplement Figure S1).

## 4 DISCUSSION

This study produced direct evidence that AGEs aid the induction of ventricular arrhythmia through increased intracellular and mitochondrial ROS, increased Na^+^_I_, and increased Ca^2+^ leaks in the RVOT, which highlights the potential role of AGEs in ventricular arrhythmogenicity in patients with DM. Burst pacing on RVOT tissue strips could induce ventricular arrhythmia after AGE 100 μg/ml infusion, which was further sustained after the administration of a superfusion of 1 μM isoproterenol; this finding represents the high propensity of patients with DM for developing ventricular arrhythmia under the influence of AGEs. The lack of ventricular arrhythmia induction in RVA after AGEs, suggesting the role of RVOT in DM related ventricular arrhythmogenesis.

Ample evidence supports the role of oxidative stress in the pathogenesis of cardiac arrhythmia (Adameova et al., 2020; Samman Tahhan et al., 2017; Yang & Dudley Jr., 2013). Increased oxidative stress in patients with DM might contribute to the damage of multiple tissues throughout the body, including the heart (Giacco & Brownlee, 2010). Consistent with previous studies (Anderson et al., 2009; Giacco & Brownlee, 2010; Hartog et al., 2007), AGE 100 μg/ml significantly increased cytosolic ROS and mitochondrial ROS of RVOT cardiomyocytes. The coexistence of increased ROS and disturbed Ca^2+^ homeostasis in this study is consistent with reports that ROS results in deleterious changes in calcium regulatory proteins and structural proteins as well as adverse cardiac remodeling (Steinberg, 2015). Moreover, AGE 100 μg/ml significantly increased the protein expression of the AGEs–RAGE and related signaling pathways, including for RAGE, NFκB P65, PKC, P38, IP3R, pIP3R, pSirt1, and Sirt1 in RVOT cardiomyocytes, suggesting their participation, at least in part, in AGE‐related electrophysiological changes in the RVOT. PKC activation is involved in several cellular responses, such as the enhancement of oxidative stress in hyperglycemia (Lien et al., 2021) and AGE, as demonstrated in this study. The modulation of PKC activity affects the dephosphorylation of the SERCA‐2 pump inhibitory protein phospholamban (PLB) and alters SR Ca^2+^ loading and Ca^2+^ transient (Braz et al., 2004). Binding AGEs to RAGE triggers signal transduction mechanisms, including the activation of NFκB and the hyperglycemia‐dependent overproduction of mitochondrial superoxide (Nishikawa et al., 2000; Schmidt et al., 1994; Yan et al., 1994). The absence of NFκB P65 favorably enhances Ca^2+^ reuptake by the SR through intracellular Ca^2+^ regulation through PLN (Zhang et al., 2013). However, the elevation of NFκB P65 in the present study lowered the reuptake of Ca^2+^ by SR. ROS are potent p38 activators, but p38 activation also drives the elevation of ROS levels, which affects the left ventricular function (Ashraf et al., 2014). Evidence indicates that intracellular Ca^2+^ overload triggered by p38 activation contributes to cardiac damage (Song et al., 2020). IP3R is a ligand‐gated calcium channel primarily localized to the SR/ER that leads to calcium release from intracellular calcium stores, and IP3 binding to the IP3R causes a conformational change (Garcia & Boehning, 2017). Together with RYR, IP3Rs also modulate cytosolic calcium levels in the heart and exert positive inotropic and arrhythmogenic effects by potentiating RYR openings (Domeier et al., 2008). Sirt1, a protein deacetylase, effectively prevents cells from oxidative stress damage (Uribarri et al., 2011). AGE‐reduced Sirt1 levels have been detected in patients with type 2 DM (T2DM), suggesting that AGEs could subdue protective mechanisms in T2DM; moreover, SIRT1 and SIRT1‐dependent NFκB P65 deacetylation levels normalized after AGE restrictions (Uribarri et al., 2011). However, we determined that Sirt1 and pSirt1 increased in AGE‐treated RVOTs, which might reflect a compensatory elevation of Sirt1 in response to an acute application of AGEs.

The prolongation of APs has consistently been detected in studies on DM (Grisanti, 2018). However, in this study, AGE 100 μg/ml did not alter the APD_90_, APD_50_, or APD_20_ of RVOTs. Although the mechanisms responsible for APD prolongation are not completely clear, K^+^ currents produce the most widely identified leading change in the hearts of patients with DM (Huang et al., 2013). However, APD prolongation and slower conduction velocity without changes were observed in altered Na^+^ and K^+^ currents in a fructose‐fat fed rat model of prediabetes (Axelsen et al., 2015). The present study revealed that only the *I*_to_ was reduced by AGE 100 μg/ml and the *I*_K1_ and *I*_Kr‐tail_ remained unchanged. Nevertheless, studies have indicated that the inhibition of AGE formation normalized prolonged APD in the ventricular muscles of patients with DM, which correlated with the restoration of both the *I*_to_ and steady‐state outward K^+^ current densities in streptozotocin‐induced DM in rats (Chang et al., 2019). Similar to our findings, no changes in APD were observed in an alloxan‐induced diabetic rabbit model (Chang et al., 2019). The discrepancies in the K^+^ current results may be related to the use of another animal species or the distinct durations of AGE application.

A reduction in conduction velocity was reported in a study on animals with diabetes (Stables et al., 2014), indicating a reduction in cell capacitance, Na^+^ channel density, or connexin. However, our study revealed no impact on the *I*_Na_; rather, AGE 100 μg/ml significantly increased the *I*_Na‐late_ in RVOT cardiomyocytes. The disparate results regarding the effect of AGE on *I*_Na_ in our study might be due to the use of an acute infusion in vitro animal model rather than a chronic in vivo animal model. Furthermore, superfusion with an *I*_Na‐late_ inhibitor, ranolazine 10 μM, abolished the induction of ventricular arrhythmia on RVOTs by burst firing and AGE 100 μg/ml superfusion, which indicates the role of *I*_Na‐late_ on DM‐related or AGE‐related ventricular arrhythmogenesis.

Cardiac Ca^2+^ homeostasis plays a vital role on cardiac function and arrhythmogenesis (Landstrom et al., 2017). Our results indicated that AGE 100 μg/ml reduced Ca^2+^_i_ transients and SR Ca^2+^ content with a prolongation of decay time in RVOT cardiomyocytes, which is inconsistent with the results of studies on animals with DM (Chang et al., 2019). Despite the reduction in Ca^2+^_i_ transients and SR Ca^2+^ content in RVOT cardiomyocytes, AGE 100 μg/ml significantly increased the SR Ca^2+^ leak. Tetracaine for inhibition of Ca^2+^ leak from ryanodine receptor (RyR) was not performed in this study. The peaks of the caffeine transients with and without tetracaine are the same because the total cellular Ca^2+^ has not changed, however, baseline Ca^2+^_i_ transient may decrease (Shannon et al., 2002). Consistent with the reduced Ca^2+^_i_ transient, AGE 100 μg/ml significantly suppressed the *I*_Ca‐L_. Studies have determined that *I*_Ca‐L_ activity is either unaltered or reduced in patients with DM (Lacombe et al., 2007; Wang et al., 1995). The diminished Ca^2+^ entry through the *I*_Ca‐L_ caused by AGE is a critical contributor to the negative effect on cardiac contractility, as reported in studies on diabetic cardiomyopathy (Lu et al., 2011). Given the increased RYR2, pRYR2 s2808, pRYR2 s2814, CaMKII, and pCaMKII and reduced SERCA2a, the SR function of AGE‐treated RVOTs is impaired. Upregulated CaMKII contributes to the connexin alterations and electrical conduction changes observed in patients with DM (Zhong et al., 2017). CaMKII affects calcium dynamics in the hearts of patients with DM and increases mitochondrial ROS, leading to increased SR calcium leaks and activation of CaMKII, thus linking oxidative stress, diabetes, and arrhythmia. Oxidized CaMKII, which is increased in people with DM and mice DM models, has been linked to ventricular arrhythmia (Luo et al., 2013; Wang et al., 2018). SERCA2a, modulated by PLB, sarcolipin, and direct phosphorylation through CaMKII, is responsible for facilitating the storage of Ca^2+^ in the SR (Frank et al., 2003). The reuptake of cytosol Ca^2+^ into the SR was reduced as SERCA2a function was suppressed. Protein kinase A phosphorylates two sites of RYR2 that are responsible for regulating the Ca^2+^ release from the SR, namely Ser2808 and CaMKII, and also phosphorylates the Ser2808 and Ser2814 sites (Ullrich et al., 2012). The increased phosphorylation by CaMKII could lead to the upregulation of RYR2, pRYR2 s2808, and pRYR2 s2814 in AGE‐treated RVOT cardiomyocytes. A hyperactive RYR‐mediated SR Ca^2+^ leak may contribute to ventricular arrhythmia (Fauconnier et al., 2011). Together with the upregulated proteins in the AGE–RAGE signaling pathway, more Ca^2+^ was released from the SR. In addition to reduced SERCA2a, the SR Ca^2+^ content was reduced because of the depletion of the Ca^2+^ store, and eventually the Ca^2+^ transient was depressed because of the reduced amount of Ca^2+^ released from the SR through RYR2. Moreover, CaMKII substantially enhanced the *I*_Na‐late_ (Hegyi et al., 2019), which increased intracellular Na^+^ loading and consequently augmented arrhythmogenesis.

When the SR function is impaired, a greater dependence on NCX for Ca^2+^ removal is expected, leading to a prominent increase in NCX function to compensate for defective SR Ca^2+^ removal at the expense of depleting the pool of Ca^2+^. NCX is electrogenic and reversible, a characteristic that depends on the prevailing electrochemical driving forces for Ca^2+^ and Na^+^, operating in either forward (Ca^2+^‐efflux) or reverse (Ca^2+^‐influx) mode. In this study, reverse NCX was augmented with AGE 100 μg/ml, primarily because of the increased *I*_Na‐late_ and cytosolic Na^+^ concentration (Na^+^_i_). In the MERLIN‐TIMI 36 study, which enrolled patients, a third of whom had DM, during the first week after admission for acute coronary syndrome, treatment with ranolazine resulted in a significantly lower incidence of an episode of VT lasting ≥4 or ≥8 beats compared with the placebo (Morrow et al., 2007). However, direct evidence of *I*_Na‐late_ inhibition, antioxidant, inhibition of AGE formation, or modulation of the AGE–RAGE signaling pathway in patients with DM for reduction of ventricular arrhythmia is lacking and warrants further study.

In conclusions, AGEs may lead to a higher rate of ventricular arrhythmia induction in RVOTs through the activation of the AGE–RAGE signaling pathway with a larger *I*_Na‐late_, Na^+^_i_, reverse mode NCX currents, increased intracellular and mitochondrial ROS, and disturbed Ca^2+^ homeostasis, which serves as a potential substrate for ventricular arrhythmogenesis and may play a role in DM‐related ventricular arrhythmogenesis.

## AUTHOR CONTRIBUTIONS

Conceptualization, Yao‐Chang Chen and Yung‐Kuo Lin; data curation, Yao‐Chang Chen, Yen‐Yu Lu and Wen‐Shiann Wu; funding acquisition, Yao‐Chang Chen, Yen‐Yu Lu, Wen‐Shiann Wu, Yung‐Kuo Lin and Yi‐Jen Chen; investigation, Yi‐Ann Chen; methodology, Yao‐Chang Chen, Yen‐Yu Lu, Yi‐Ann Chen and Yung‐Kuo Lin; project administration, Yi‐Jen Chen; resources, Shih‐Ann Chen; supervision, Yung‐Kuo Lin; visualization, Shih‐Ann Chen and Yi‐Jen Chen; writing‐original draft, Yung‐Kuo Lin; writing‐review & editing, Yao‐Chang Chen and Yung‐Kuo Lin. All authors have read and agreed to the published version of the manuscript.

## CONFLICT OF INTEREST

The authors have declared that no conflict of interest exists.

## ETHICS STATEMENT

The animal study was reviewed and approved by Institutional Animal Care and Use Committee (IACUC) National Defense Medical Center.

## Supporting information


Supplemental Figure 1
