# Supplementary material for: Advanced glycation end products modulate electrophysiological remodeling of right ventricular outflow tract cardiomyocytes: A novel target for diabetes‐related ventricular arrhythmogenesis
Source: Physiol Rep. 2022 Nov 2;10(21):e15499. doi: 10.14814/phy2.15499 (PMC9630757; doi:10.14814/phy2.15499)
Supplement: Supplementary file 1 — Supplemental Figure 1 [file PHY2-10-e15499-s001.pdf]

Supplemental Figure 1

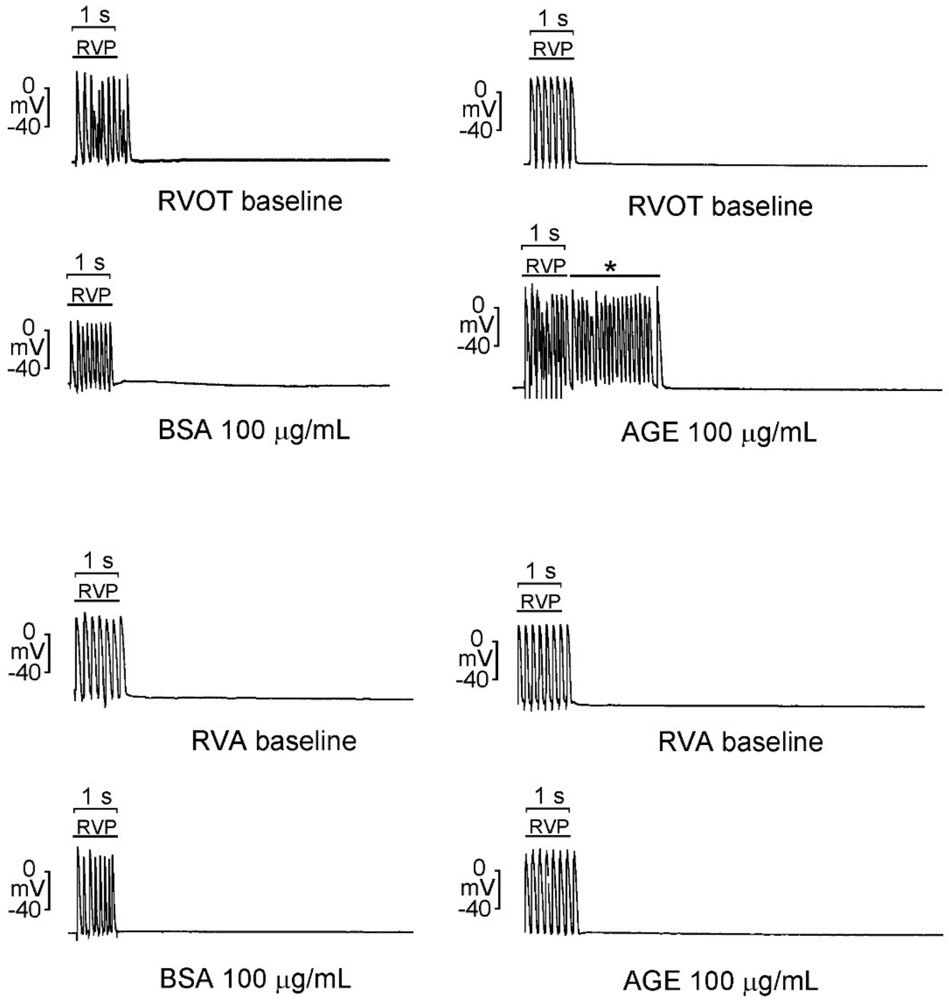

Supplemental Figure 1. Effects of AGEs on the ability of RVOT or RVA to induce ventricular arrhythmia. Upper panel Examples of burst pacing on RVOT at baseline, after bovine serum albumin, and after AGE 100 μg/mL (N = 7). Non-sustained ventricular arrhythmia was noted in RVOT after AGE incubation only. Lower panel: Examples of burst pacing on RVA at baseline, after bovine serum albumin, and after AGE 100 μg/mL (N = 5). N, animal number.
